# Correction using Halo Gravity Traction for Severe Rigid Neuromuscular Scoliosis: A Report of Three Cases

**DOI:** 10.5704/MOJ.1903.010

**Published:** 2019-03

**Authors:** JO Mejabi, OM Sergeenko, SO Ryabykh

**Affiliations:** Department of Surgery, Federal Medical Centre, Birnin Kebbi, Nigeria; * Department of Orthopaedics, Russian Ilizarov Scientific Center, Kurgan, Russia

**Keywords:** neuromuscular scoliosis, halo gravity traction

## Abstract

Severe rigid neuromuscular scoliosis is a major challenge to the spine surgeon due to the possibilities of neurological sequelae from acute correction of the deformity. Halo gravity traction has been considered as a way of reducing the deformity before correction to prevent neurological complications. Three female patients with severe neuromuscular scoliosis aged seven to 13 years with main coronal Cobb angle of 95°-128° and Kyphotic Cobb of 47°-118° having ≤35% flexibility on traction, had between 18 to 23 days of 16 hour/day of halo gravity traction and night time supine traction with 4kg weight for 7-8 hours. They had 28.9% and 18.5% of main coronal and kyphotic Cobb angle correction post-traction respectively. All had posterior instrumentation and post-operatively, they had correction of main coronal Cobb angle of 29°-58° and kyphotic Cobb angle of 30°-77° with no neurological complication. Halo gravity traction is therefore a viable option for reducing post-operative neurological complication in rigid severe scoliosis.

## Introduction

Managing severe rigid deformities in neuromuscular scoliosis has always been a major challenge to spine surgeons. Surgery is done in these patients to stop the progression of a disabling deformity, reduce the size of the curve and restore trunk balance. This ultimately leads to improved quality of life. Current surgical practice includes anterior and posterior column release and osteotomies to correct the deformity and this improves the physical appearance of the patient but is fraught with neurologic complications which may worsen the quality of life of the patient.

With improvement in the correction of deformities using anterior and posterior release with spinal instrumentation, there is a need to find an alternative method of reducing the curse before surgery which may help to reduce neurologic compromise and increase the quality of life of the patient. If the patient is subjected to peri-operative traction, this may reduce the extent of surgery needed and allow for a better overall correction of severe rigid deformities with fewer neurologic complications. Halo gravity traction has been considered as a way of reducing the deformity before surgery to prevent neurological complications^1^. We report the surgical and radiological outcome of severe paediatric kypho-scoliosis in three patients treated with three weeks of pre-operative halo gravity traction followed by fusion. Different time frames have been used by various authors in literature ranging from four weeks to more than three months for traction before surgery. However, we decided to use three weeks or less of halo gravity traction for the patients to see whether we could get similar result.

## Case Report

We report the cases of three female patients with neuromuscular scoliosis treated in the Pediatric Spine unit of Ilizarov Centre. The three patients had severe rigid curves. Patient parameters evaluated included personal data (age, sex, height, diagnosis), pre-operative data (traction duration), peri-operative data (type of surgery, operative time, blood loss, instrumentation, complications, hospital stay), clinical data (SRS-24 questionnaire before surgery and at six months after surgery, pre-operative and post-operative height) and radiographic data prior to traction, after traction and after surgery (of major curve coronal Cobb angle, secondary curve Cobb angle, regional kyphosis angle and, loss of correction). In addition, patients were evaluated for complications. Radiographic studies included pre-operative and post-operative standing antero-posterior and lateral radiographs.

Six to eight halo pins were placed for each of the patients. The halo was placed resting slightly below the equator of the skull just above the eyebrows and the cephalad portion of the earlobes. Traction was carried out on halo-gravity, in the vertical position of the patients, under their own weight. Patients were carefully monitored with daily full neurologic examination and instructed to immediately report any unusual symptoms. They were hospitalised during the traction under constant supervision of medical staff.

Mean age was 10.7 years (7 to 13 years). The three had severe pre-traction scoliosis with mean main coronal Cobb angle of 109.7° (95°-128°), mean regional Kyphosis Cobb angle of 84.7° (47°-118°). The pre-operative mean height was 121cm (102-134cm) (Fig. 1a and d, 2a and d, 3a and d). They all had ambulatory traction with walking frame for 16 hours in a day with the weight acting against gravity to reduce the Cobb angle (Fig. 1b and e, 2b and e, 3b and e) and at night seven to eight hours on traction in bed with 4 kg weight to continue the traction. They had rigid scoliosis with flexibility not more than 35% even in traction (Fig. 1e, 2e, 3e). The number of days that each patient was on traction was recorded with the Cobb angles before and after completion of traction (Table I) (Fig. 1,2,3). Post-traction pre-operative mean main coronal Cobb angle was 78° (59°-108°) and mean regional Kyphosis of 69° (4°-105°). The percentage correction after traction was 28.9% for main coronal Cobb angle and 18.5% for Kyphosis Cobb angle in the three patients.

**Table I:** Operative- and post-operative clinical and radiologic data of the three patients with neuromuscular scoliosis

Patient (Sex/Age)	Height (cm)	Diagnosis	MC Cobb (degree)	Regional kyphosis Cobb (degree)	Traction duration (days)	Post-tr MC Cobb (degree)	Post-tr Regional kyphosis Cobb (degree)	Post-op MC Cobb (degree)	Post-op Regional kyphosis Cobb (degree)	Post-op height (cm)	Loss of correction MC (%)	Loss of correction Regional kyphosis (%)
Patient 1 (Female/7)	102	Undifferentiated connective-tissue dysplasia	95	89	20	59	62	29	36	110	0	0
Patient 2 (Female/13)	134	Ehlers-Danlos syndrome	128	118	18	108	105	58	77	142	7	3
Patient 3 (Female/12)	127	Neuro-fybromatosis	106	47	23	67	40	38	30	133	0	0

MC=major curve; Post-tr=post traction; Post-op=post-operative

**Fig. 1:**
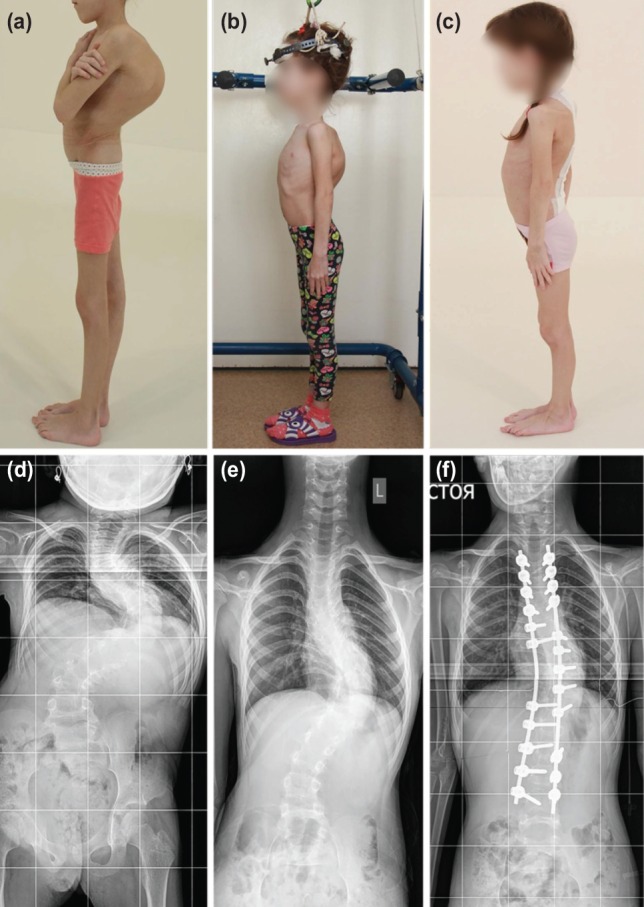
7-year old patient with undifferentiated connective-tissue dysplasia (Case 1). Images (a) pre-operative, (b) on traction and (c) post-operative. Radiographs (d) pre-operative, (e) on traction and (f) post-operative.

**Fig. 2:**
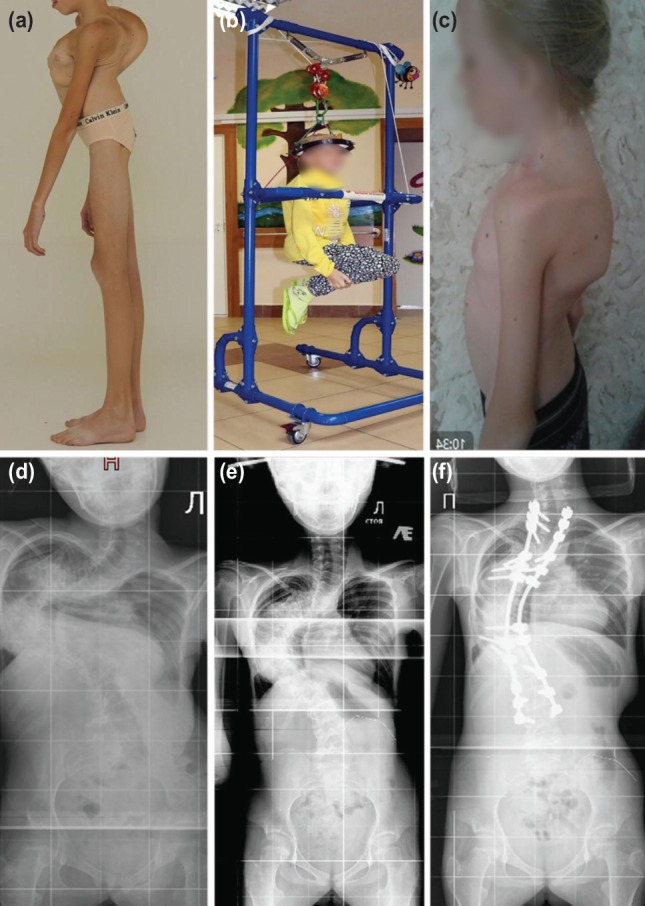
13-year old with Ehlers-Danlos syndrome (Case 2). Images (a) pre-operative, (b) on traction and (c) post-operative. Radiographs (d) pre-operative, (e) on traction and (f) post-operative.

**Fig. 3:**
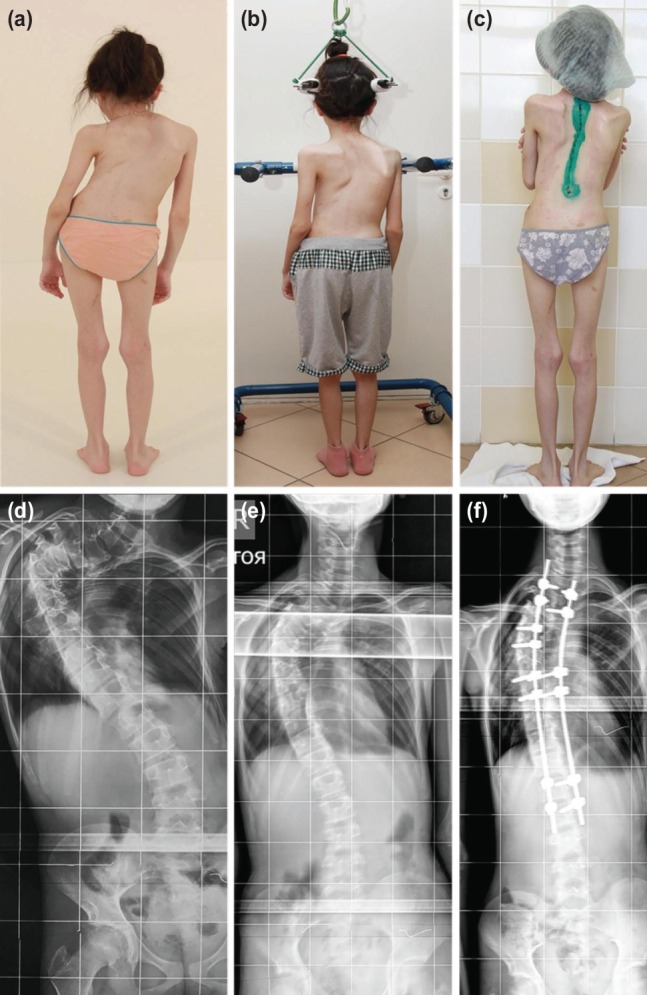
13-year old with Neurofibromatosis (Case 3). Images (a) pre-operative, (b) on traction and (c) post-operative. Radiographs (d) Pre-operative, (e) on traction and (f) post-operative.

They all underwent posterior spinal fusion. The first patient had it from T3 to L4, the second from T2 to L2 and third from T1 to L3 (Table I). Mean blood loss was 323.3mls (170-600mls) and surgery time was 265mins (195-365mins). The first and the third patients stayed for nine days after surgery before discharge while the second patient stayed for eight days. Their post-operative recovery period was uneventful (Fig. 1f, 2f and 3f). Mean pre-operative SRS-24 was 2.9 (2.5-3.4) while at six months post-operatively, it was 3.6 (2.9-4.1).

## Discussion

Rigid severe scoliosis is a serious challenge to the surgeons due to the possible neurological complications associated with the correction. Halo gravity traction has been used to progressively reduce the severity of the scoliotic angle in order to prevent neurological complications that can arise as a result of aggressive surgical correction. The fear of possible neurological complications has led to reduction in the extent of surgical procedure to correct the deformity with the result that the correction of the pre-operative Cobb angle was more conservative. It had also led to the avoidance of anterior release surgery with its attendant complications^1^.

The percentage correction after traction was 28.9% for main coronal Cobb angle and 18.5% for Kyphosis Cobb angle in the three patients. This was similar to the report in 2000 by Janus *et al* of 20 patients with Osteogenesis Imperfecta with 32% correction for scoliosis and 24% correction for kyphosis after traction^2^. All the three patients in our current report had posterior release and instrumentation. Post-operatively, there was improvement in the patients’ parameters with mean main coronal Cobb angle of 41.7°, mean Kyphosis Cobb of 47.7°. The height increased to 128.3cm. There was loss of 7° in main coronal Cobb angle and 3° in Kyphosis Cobb angle in one of the patients. Previous studies as quoted by Li *et al* had improvement in the main Cobb angle and Kyphotic angle among their subjects while the same Li *et al* in their own study had increase in patients’ height even with halo gravity traction alone^3^.

Koptan *et al* compared patients with traction to the ones without traction and achieved better correction, shorter operative time and similar blood loss compared to those without preoperative traction^4^. None of the patient had neurological complication from the correction or the surgery itself. Another study in their meta-analysis of halo-gravity traction in the treatment of severe spinal deformity found that partial correction could be achieved preoperatively with halo-gravity traction, and it may help decrease aggressive procedures and reduce neurologic complications. However, traction could not increase preoperative flexibility or final correction. Traction-related complications, although usually not severe, were not rare^5^. The limitation of our report is that it is study of only three patients. A prospective study with a large number of patients is needed to make objective conclusions about halo gravity traction.

Halo gravity traction pre-operatively is a simple and safe method of reducing neurological complication and preventing extensive anterior release in severe rigid neuromuscular scoliosis while achieving comparable results in terms of correction of deformity and improving physical appearance.
